# Unfolded Protein Response and Activated Degradative Pathways Regulation in GNE Myopathy

**DOI:** 10.1371/journal.pone.0058116

**Published:** 2013-03-05

**Authors:** Honghao Li, Qi Chen, Fuchen Liu, Xuemei Zhang, Wei Li, Shuping Liu, Yuying Zhao, Yaoqin Gong, Chuanzhu Yan

**Affiliations:** 1 Department of Neurology, Qilu Hospital of Shandong University, Jinan, China; 2 Department of Neurology, Provincial Hospital Affiliated to Shandong University, Jinan, China; 3 Department of Neurology, Yantai Yuhuangding Hospital, Yantai, China; 4 Department of General Internal Medicine, Shandong University Hospital, Jinan, China; 5 Key Laboratory for Experimental Teratology of the Ministry of Education and Institute of Medical Genetics, School of Medicine, Shandong University, Jinan, China; University of Queensland, Australia

## Abstract

Although intracellular beta amyloid (Aβ) accumulation is known as an early upstream event in the degenerative course of UDP-N-acetylglucosamine 2-epimerase/N-acetylmannosamine kinase (GNE) myopathy, the process by which Aβdeposits initiate various degradative pathways, and their relationship have not been fully clarified. We studied the possible secondary responses after amyloid beta precursor protein (AβPP) deposition including unfolded protein response (UPR), ubiquitin proteasome system (UPS) activation and its correlation with autophagy system. Eight GNE myopathy patients and five individuals with normal muscle morphology were included in this study. We performed immunofluorescence and immunoblotting to investigate the expression of AβPP, phosphorylated tau (p-tau) and endoplasmic reticulum molecular chaperones. Proteasome activities were measured by cleavage of fluorogenic substrates. The expression of proteasome subunits and linkers between proteasomal and autophagy systems were also evaluated by immunoblotting and relative quantitative real-time RT-PCR. Four molecular chaperones, glucose-regulated protein 94 (GRP94), glucose-regulated protein 78 (GRP78), calreticulin and calnexin and valosin containing protein (VCP) were highly expressed in GNE myopathy. 20S proteasome subunits, three main proteasome proteolytic activities, and the factors linking UPS and autophagy system were also increased. Our study suggests that AβPP deposition results in endoplasmic reticulum stress (ERS) and highly expressed VCP deliver unfolded proteins from endoplasmic reticulum to proteosomal system which is activated in endoplasmic reticulum associated degradation (ERAD) in GNE myopathy. Excessive ubiquitinated unfolded proteins are exported by proteins that connect UPS and autophagy to autophagy system, which is activated as an alternative pathway for degradation.

## Introduction

Distal myopathy with rimmed vacuoles (DMRV) or GNE myopathy is an autosomal recessive disorder clinically characterized by adult-onset of muscle weakness starting from distal muscles of legs and slowly progressing to proximal musculature with relative quadriceps sparing [1], [2]. Typical pathological changes of GNE myopathy include rimmed vacuoles and cytoplasmic and/or nuclear filamentous inclusions. GNE myopathy is caused by mutations in the *GNE* gene (GenBank Accession No. NP_005467.1) [3], [4] that encodes the bifunctional enzyme UDP-N-acetylglucosamine 2-epimerase/N-acetylmannosamine kinase that catalyzes two critical steps in sialic acid synthesis [5]. Sialic acid is the terminal sugar on glycoconjugates and is involved in several cellular processes [6]. *GNE* mutations lead to decreased GNE/MNK enzymatic activities and reduced production of sialic acid [7]. Although excellent experimental works have been done in pursuit of the supportive evidence for the hypothesis that it is the decrease in intracellular sialic acid content that leads to the muscle degeneration in GNE myopathy, the exact cellular mechanisms behind the development of the myopathy have remained elusive [8]–[10]. Intracellular Aβ deposition in myofibers was rare and only documented in a few muscle diseases, such as sporadic inclusion body myositis (sIBM) and GNE myopathy [11]. Pathological features of GNE myopathy are very similar to those of sIBM. Both have similar intracellular deposits that are congophilic and immunoreactive to AβPP, Aβ, p-tau and other proteins related to ERS. The role of amyloid deposition in the pathogenesis of muscle diseases is highlighted in a sIBM mouse model, in which a correlation of intracellular amyloid levels and motor weakness was seen [12]. But how intracellular Aβ accumulation contributes to the progressive degeneration of GNE myopathy muscle is not fully understood. In this study we investigated the deposition of AβPP and the secondary responses in GNE myopathy such as ERS and proteosomal activation. We also studied the linkers between UPS and autophagy system and tried to find clues to the pathophysiology of GNE myopathy.

## Materials and Methods

### 1 Ethics Statement

This study was conducted according to the principles expressed in the Declaration of Helsinki and approved by the Medical Ethics Committee of Shandong University and all participants were included after informed consent for the collection of samples and subsequent analyses.

### 2 Muscle Biopsies

All muscle specimens were snap frozen in cooled isopentane, and stored at −80°C until analysis. All GNE myopathy patients were clinically and genetically diagnosed Mongoloid individuals who visited the Peripheral Neuropathy and Myopathy Department of Qilu Hospital (Table S1). The method of *GNE* gene sequencing was performed as described earlier and partial results (patient 1–6) have been reported [13]. The control muscle specimens were collected from individuals who had undergone muscle biopsy and were eventually diagnosed as normal. We matched appropriate control sample to GNE myopathy sample, by age, gender and muscle type, as closely as possible.

### 3 Immunofluorescence Microscopy

Immunofluorescence (IF) was performed as follows: 7-µm thick transverse freshly frozen sections were fixed in ice-cold acetone at 4°C for 10 min after air drying at room temperature for 15 min. Sections were washed in 0.01 M phosphate-buffered saline (PBS) with a PH of 7.4, and preincubated in 10% normal goat serum for 30 min. They were then incubated overnight at 4°C with well-characterised antibodies (Table S2), Following 3×2-min washing in PBS, the sections were incubated with rhodamine labeled secondary anti-mouse antibody diluted 1∶200 or fluorescein isothiocyanate labeled secondary anti-rabbit antibody diluted 1∶200 in 2% BSA for 1 h. Following 3×2-min washing in PBS and 3×2-min washing in distilled water, the sections were sealed with 0.1 M soda-sodium bicarbonate glycerol buffer with a PH of 7.2 and observed under fluorescence microscope (DP71, Olympus, Japan). To block nonspecific binding of antibody to Fc receptors, sections were pre-incubated with normal goat serum diluted 1∶10. Omission of the primary antibody was used as controls for staining specificity.

### 4 Immunoblotting

Western blotting analysis was performed. Briefly, about 50 pieces of 10-µm-thick sections of frozen muscle were collected at −25°C and rapidly homogenized on ice with cell lysis buffer for western and IP kits (P0013, Beyotime China). Protein concentration was measured by the BCA method (Applygen Technologies Inc, Beijing, China). Twenty to sixty micrograms of protein was loaded on a 10%–12% polyacrylamide gel, separated by electrophoresis, and then transferred to a nitrocellulose membrane. Nitrocellulose membranes were blocked in 5% (w/v) blocking reagent in PBS plus 0.1% Tween 20, and were incubated overnight at 4°C with one of the primary antibodies (Table S2). After being washed, the membranes were incubated with secondary antibody conjugated to horseradish peroxidase. The blots were developed using an enhanced chemiluminescence system. Protein loading was evaluated by α-skeletal actin or GAPDH band visualized with a monoclonal antibody. Statistical analyses were performed by a one-tailed t-test. Significance level was set at P<0.05. Data are reported as means ± SD for all groups.

### 5 Measurement of Proteasome Activity

Three main proteasome activities were determined by evaluating the cleavage of specific fluorogenic substrates [14]. Muscle samples from four patients with GNE myopathy and four age-matched controls were homogenized in 20 mmol/L Tris-HCl (Sigma-Aldrich, America), pH 7.2, containing 0.1 mmol/L ethylenediamine tetraacetic acid (Sigma-Aldrich, America), 1 mmol/L 2-mercaptoethanol (Sigma-Aldrich, America), 5 mmol/L ATP(Sigma-Aldrich, America), 20% glycerol (Fine chemical plant, Laiyang, China) and 0.04% (v/v) Nonidet P-40 (ST366, Beyotime, China), centrifuged, the supernatant collected, and protein concentration determined using the BCA method. Subsequently, 200 µg of biopsied muscle protein were incubated in 100 µmol/L fluorogenic substrates for the following three protease activities: Z-Leu-Leu-Glu-AMC (substrate II) for peptidyl-glutamyl peptide-hydro (PGPH); Suc-LLVY-AMC (substrate III) for chymotrypsin-like (CTL); and Z-Val-Lys-Met-AMC (substrate IV) for trypsin-like (TL) activity (Merk4 Biosciences, China). Fluorescence emission was excited at 360 nm and recorded at 430 nm. Statistical analyses were performed by a one-tailed t-test for the proteasome activity. Significance level was set at P<0.05. Data are reported as means ± SD for all groups.

### 6 Relative-quantitative Real-time RT-PCR

Total RNA was isolated from frozen muscle biopsy tissues using TRIzol reagent (Tiangen, China) according to manufacturer’s protocol. The concentration of total RNA was measured by a spectrophotometry and reverse-transcribed with a RT-PCR kit (Tiangen, China). The real-time PCR was performed using the SYBR Green I kit (Tiangen, China). All the primers are listed in Table S3. The specificity of products generated for each set of primers was examined with a single melting curve and gel electrophoresis. The relative expression levels of each targeted gene were normalized by subtracting the corresponding beta-actin threshold cycle (CT) values by the ΔΔC_T_ comparative method. A total of four samples for each group were used, and each sample was run in triplicate. All the results are expressed as means ± SD. The individual groups were tested for differences by using one-way ANOVA repeated measurements, followed by independent samples t-test. Significance level was set at P<0.05.

## Results

### 1 Abnormal Protein Aggregates Accumulation

**Figure 1:**
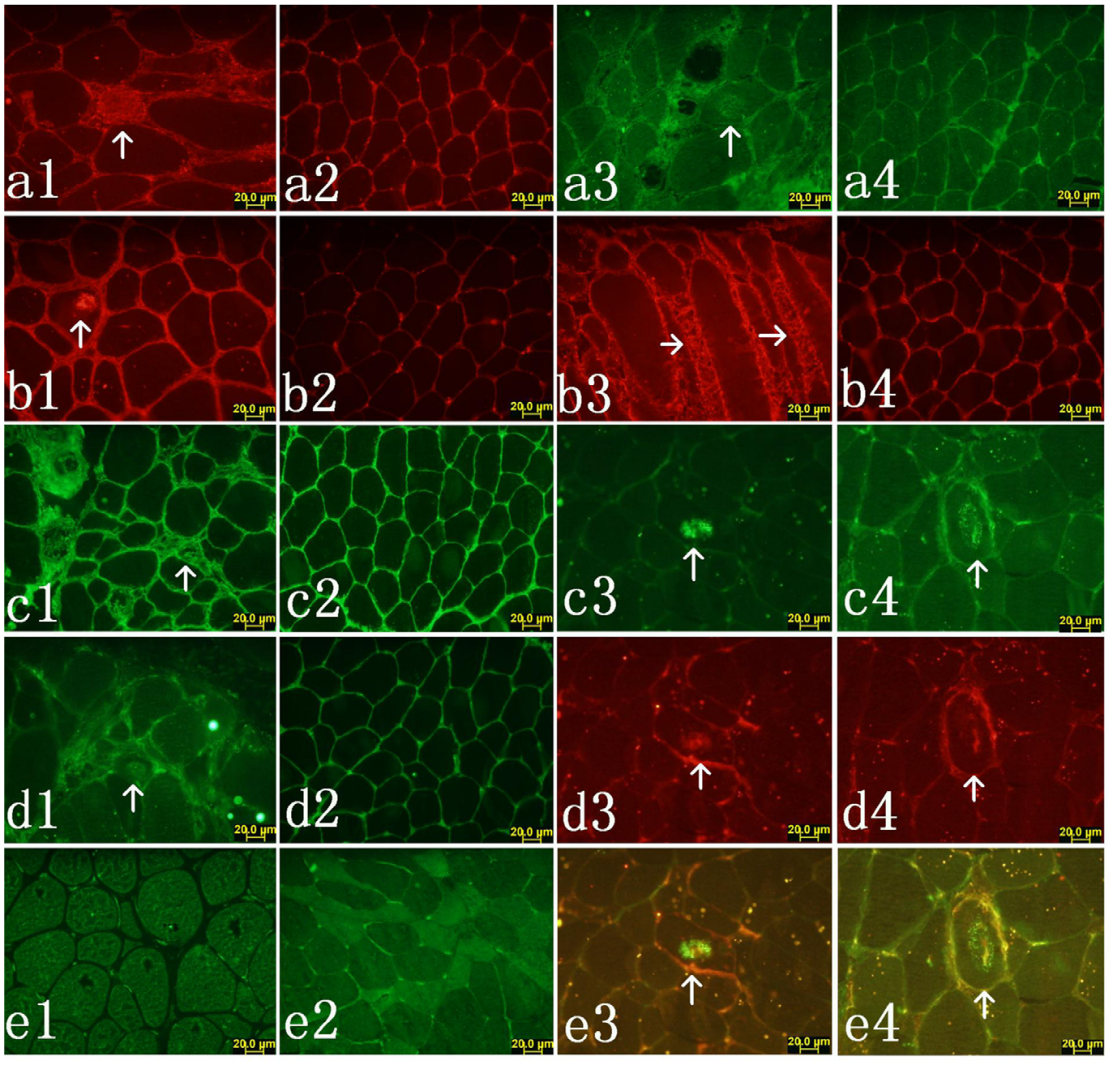
Immunofluorescence shows AβPP and p-tau aggregates and molecule chaperones in GNE myopathy muscle. Single-label immunofluorescence illustrates strongly immunoreactive aggregates of AβPP (a1) in GNE myopathy, nonspecific staining in muscle interstitial of normal control (a2), aggregates of p-tau (a3) in GNE myopathy and nonspecific staining in muscle interstitial of normal control (a4). Single-label immunofluorescence illustrates strongly immunoreactive aggregates of calnexin (b1) in GNE myopathy, nonspecific staining in muscle interstitial of normal control (b2), aggregates of calreticulin in GNE myopathy (b3), nonspecific staining in muscle interstitial of normal control (b4), aggregates of GRP94 in GNE myopathy (c1), nonspecific staining in muscle interstitial of normal control (c2) and aggregates of GRP78 in GNE myopathy (d1) and nonspecific staining in muscle interstitial of normal control (d2). No positive staining of ERp72 was found in cytoplasm of GNE myopathy muscle fibers (e1) and normal control muscle fiber (e2). Double-label immunofluorescence illustrates that in GNE myopathy muscle fibers the aggregates immunoreactive for GRP94 (c3) are also immunoreactive for AβPP (d3) (e3 merged). Aggregates immunoreactive for GRP78 (c4) are also immunoreactive for AβPP (d4) (e4 merged). b1, d1 and e1 were from sample HIBM-1; c3, d3, e3, c4, d4 and e4 were from sample HIBM-2; c1 and b3 were from sample HIBM-3; a1 and a3 were from sample HIBM-5; a2 and a4 were from control-1; b2, d2 and e2 were from control-2; c2 and b4 were from control-3.

**Figure 2:**
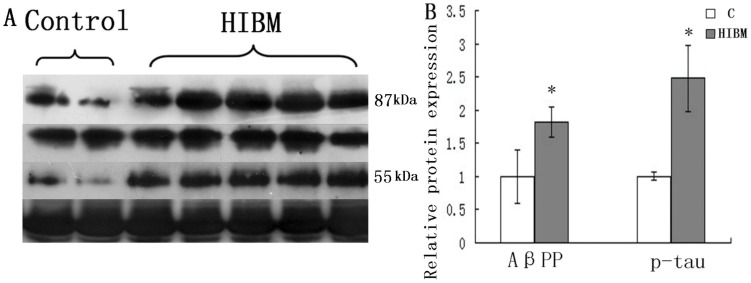
Immunoblots of AβPP and p-tau in control and GNE myopathy muscle biopsies. A: Immunoblots of muscle homogenates of two normal control and five GNE myopathy muscle biopsies demonstrate in GNE myopathy a much stronger expression of AβPP and p-tau as compared to control muscle biopsies. C = control tissue, n = 2 (Samples are from control-1 and control-2); GNE myopathy, n = 5 (Samples are from GNE myopathy -1, -2, -3, -4, and -5). B: Densitometric analysis of the blots in A performed using Quantity One. Protein loading was evaluated by the actin band. Data are indicated as mean±SD. Significance was determined by one-tailed unpaired t-test. The level of significance was set at P<0.05. The densitometry graphs are representative of only the one chosen in the corresponding blots.

In GNE myopathy muscle biopsies, well-defined round aggregates that were immunoreactive to antibodies against AβPP were found in the cytoplasm (Fig. 1a1). Appearance of p-tau aggregates was similar to AβPP aggregates, as demonstrated by immunofluorescence microscopy (Fig. 1a3). Nonspecific staining was only observed in the interstitial substance of muscle fibers from normal control (Fig. 1a2 and a4). Levels of AβPP and p-tau were increased in GNE myopathy muscle biopsies on immunoblots (Fig. 2A). In five GNE myopathy muscle biopsies, densitometry of AβPP and p-tau after normalization to the corresponding actin band showed, that AβPP was increased by 82.7% and p-tau by 148.1% (P<0.05) as compared with the age-matched control muscle biopsies (Fig. 2B).

### 2 Upregulation of Molecular Chaperones

**Figure 3:**
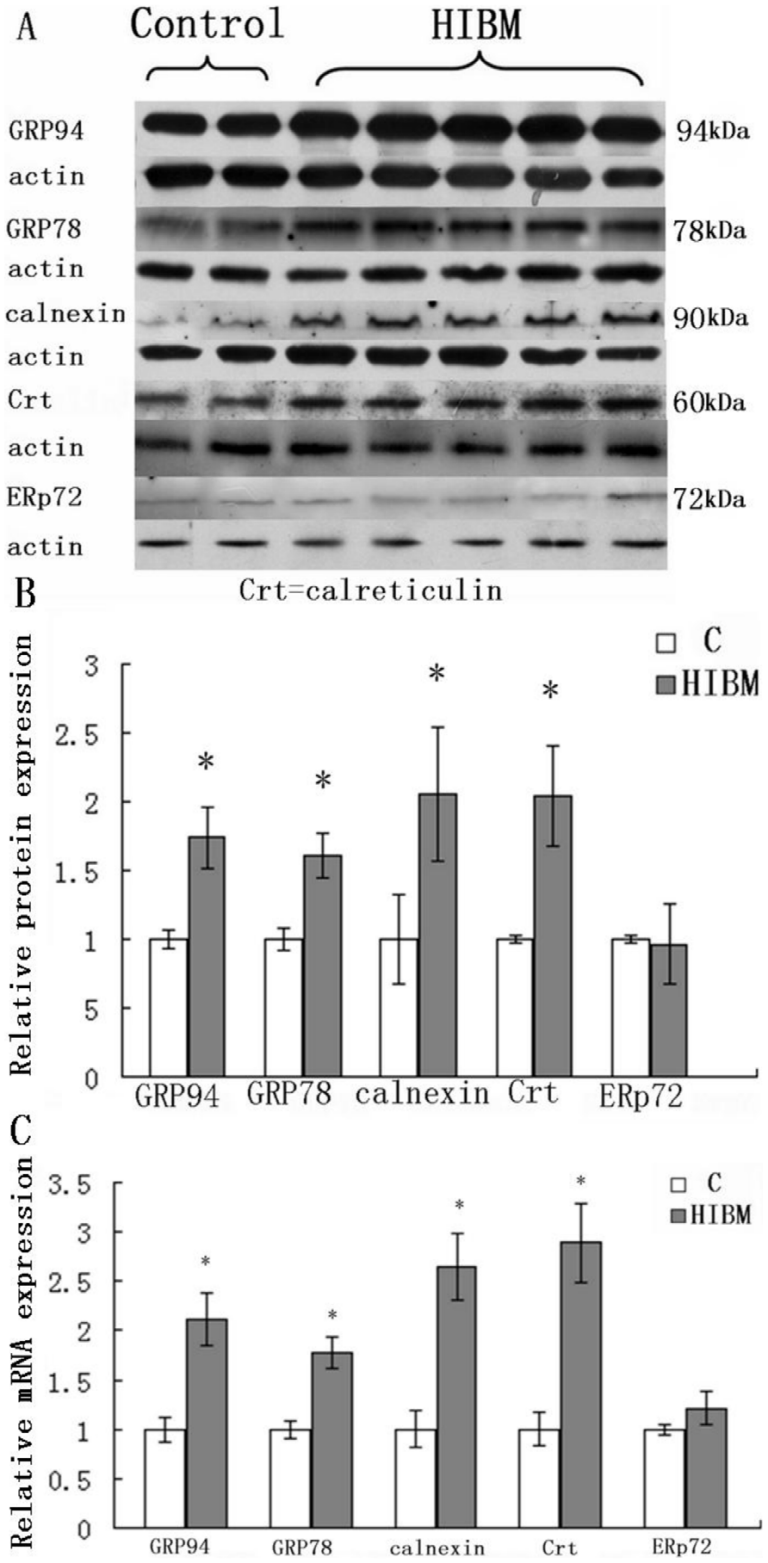
Immunoblots and relative mRNA levels of of endoplasmic reticulum molecular chaperones in normal control and GNE myopathy muscle biopsies. A: Immunoblots of muscle homogenates of two normal control and five GNE myopathy muscle biopsies demonstrate in GNE myopathy a much stronger expression of GRP94, GRP78, calnexin and calreticulin except for ERp72 as compared to control muscle biopsies. C = control tissue, n = 2 (Samples are from control-1 and -2); GNE myopathy, n = 5 (Samples are from HIBM2-1, -2, -3, -4, and -5) B: Densitometric analysis of the blots in A performed using Quantity One. Protein loading was evaluated by the actin band. Data are indicated as mean±SD. Significance was determined by one-tailed unpaired t-test. The level of significance was set at P<0.05.C: Relative mRNA levels of molecular chaperones in normal and GNE myopathy patient biopsies. A total of four samples for each group were used, and each sample was run in triplicate for real-time PCR. C = control tissue, n = 4 (Samples are from control-1, -3, -4 and -5); GNE myopathy, n = 4 (Samples are from HIBM-5, -6, -7 and -8). Relative mRNA levels of four molecular chaperones were significantly increased in GNE myopathy muscle biopsies, GRP94 to112.3%, GRP78 to78.1%, calnexin to165.5%, and calreticulin to 189.6%. Data are shown as means ± SD. The level of significance was set at P<0.05. The densitometry graphs are representative of only the one chosen in the corresponding blots.

Several important molecular chaperones localized in endoplasmic reticulum, GRP94, GRP78, calreticulin, calnexin and endoplasmic reticulum protein 72 (ERp72), have been used extensively as indicators for ERS. Immunofluorescence study showed that calnexin (Fig. 1b1), calreticulin (Fig. 1b3), GRP94 (Fig. 1c1), GRP78 (Fig. 1d1) were expressed in the muscle tissues of GNE myopathy patients whereas, normal control exhibited no immunoreactivity for these molecules (Fig. 1b2, b4, c2, and d2). There were no aggregates immunoreactive to ERp72 in either GNE myopathy (Fig. 1e1) or normal control (Fig. 1e2). Double-label fluorescence immunohistochemistry, showed that inclusions immunoreactive to GRP94 and GRP78 co-localized with AβPP in some muscle fibers (Fig. 1c3–e3; c4–e4). Production of those molecules, except for ERp72, by immunoblotting was elevated in GNE myopathy muscle biopsies (Fig. 1A). Densitometry analysis showed that GRP94 was increased by 73.8%, GRP78 by 61.2%, calnexin by 105.3%, and calreticulin by 104.2% (P<0.05) (Fig. 3B). There was no difference in the expression of ERp72 between GNE myopathy patients and normal controls on either immunofluorescence microscopy or immunoblotting. Furthermore, real-time RT-PCR analysis demonstrated the similar trend of molecular chaperones overexpression on mRNA level (Fig. 3C). All these findings suggested UPR in muscle tissue GNE myopathy patients.

### 3 Upregulation of UPS Components and Activities

**Figure 4:**
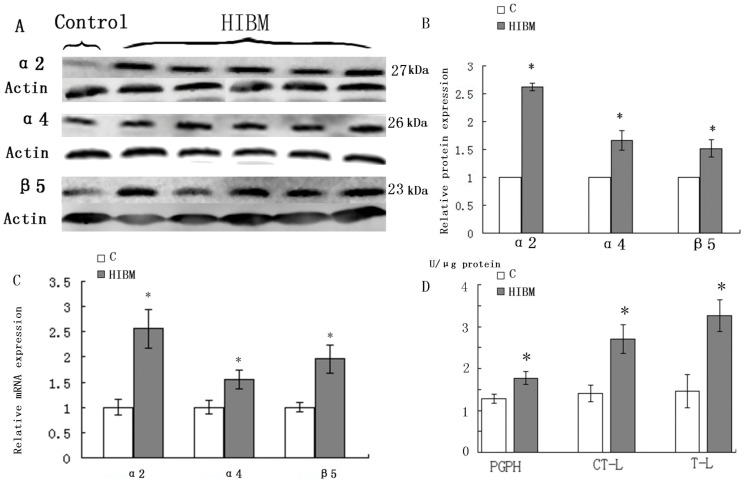
Immunoblots, relative mRNA levels of 20S proteasome subunits α2, α4, and β5 and proteasome enzymatic activities in control and GNE myopathy muscle biopsies. A: Immunoblots of muscle homogenates of normal control and GNE myopathy muscle biopsies demonstrate in GNE myopathy a much stronger expression of α2, α4 and β5 as compared to control muscle biopsies. C = control tissue, n = 1 (Sample is from control-2); GNE myopathy, n = 5 (Samples are from HIBM-2, -4, -5, -6 and -8) B: Densitometric analysis of the blots in A performed using Quantity One. Protein loading was evaluated by the actin band. Data are indicated as mean±SD. Significance was determined by one-tailed unpaired t-test. The level of significance was set at P<0.05. C: Relative mRNA levels of 20S proteasome subunits α2, α4, and β5 in normal and GNE myopathy patient biopsies. A total of four samples for each group were used, and each sample was run in triplicate for real-time PCR. C = control tissue, n = 4 (Samples are from control-1, -3, -4 and -5); GNE myopathy, n = 4 (Samples are from HIBM-5, -6, -7 and -8). Relative mRNA levels of three 20S proteasome subunits were significantly increased in GNE myopathy muscle biopsies, α2 to156.4%, α4 to 56.7%, and β5 to 96.3%. Data are shown as means ± SD. The level of significance was set at P<0.05.D: Proteasome enzymatic activities in control and GNE myopathy muscle biopsies. Proteasome trypsin-like (TL), chymotrypsin-like (CTL), and peptidyl-glutamyl peptide-hydro (PGPH) activities were measured in four GNE myopathy and four normal control muscle biopsies and results were normalized to total protein quantity in the same muscle biopsies. C = control tissue, n = 4 (Samples are from control-1, -3, -4 and -5); GNE myopathy, n = 4 (Samples are from HIBM-5, -6, -7 and -8). All three proteasome activities are significantly increased in GNE myopathy muscle biopsies, CTL by 48.2%, TL by 55.3%, and PGPH by 27.5% (P<0.05) of control muscle biopsies. Data are indicated as mean±SD. Significance was determined by the one-tailed unpaired t-test. The level of significance was set at P<0.05.

The expression of 20S proteasome subunits α2, α4 and β5 was increased in GNE myopathy muscles on both transcript and protein levels (Fig. 4A, C). Western blots densitometry showed that α2 was increased by 162.3%, α4 by 66.3% and β5 by 51.7% (P<0.05) (Fig. 4B). 20S proteasome activities were measured and normalized to protein value measured in each patient. In GNE myopathy biopsies, chymotrypsin-like (CTL), trypsin-like (TL), and peptidyl-glutamyl peptide-hydrolyzing (PGPH) protease activities were increased by 48.2%, 55.3%, and 27.5% (P<0.05), respectively compared with the control biopsies (Fig. 4D).

### 4 Upregulation of VCP and Linkers between UPS and Autophagy System

**Figure 5:**
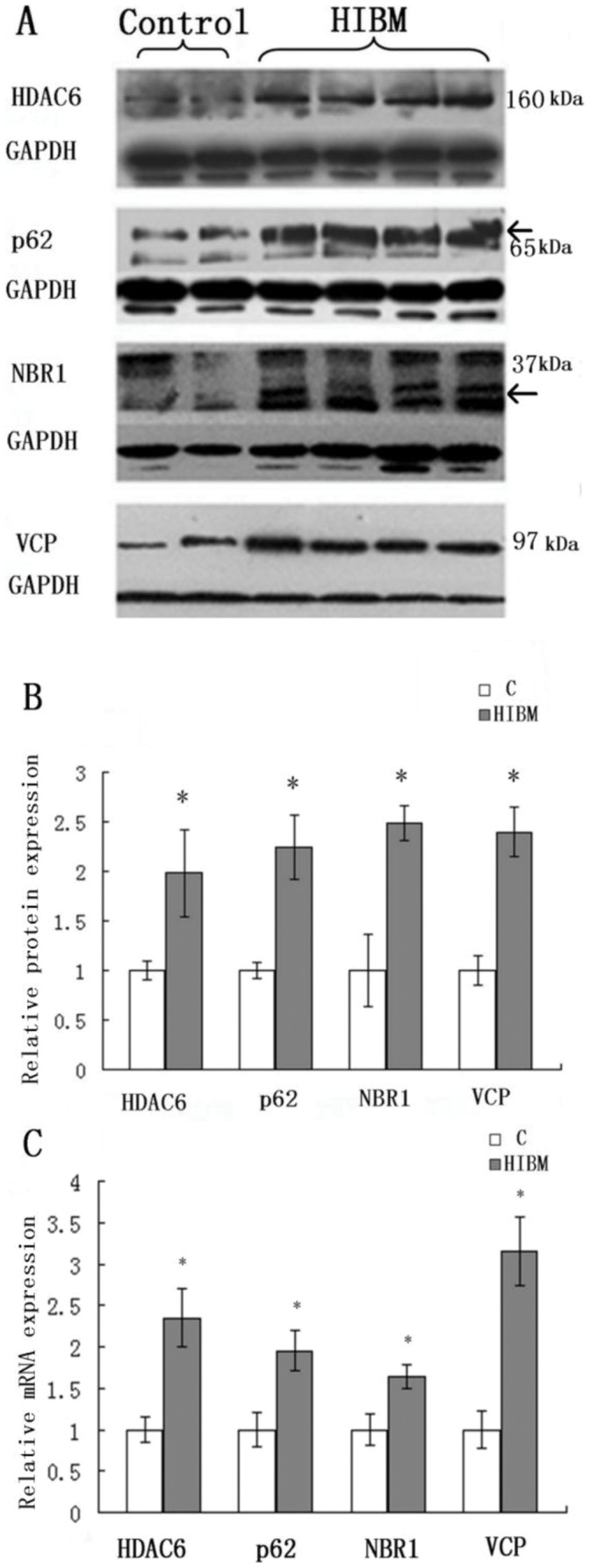
Immunoblots and relative mRNA levels of VCP and linkers between UPS and autophagy in control and GNE myopathy muscle biopsies. The densitometry graphs are representative of only the one chosen in the corresponding blots. A: Immunoblots of muscle homogenates of normal control and GNE myopathy muscle biopsies demonstrate in GNE myopathy a much stronger expression of HDAC6, p62, NBR1 and VCP as compared to control muscle biopsies. C = control tissue, n = 2 (Samples are from control-4 and -5); GNE myopathy, n = 5 (Samples are from HIBM-4, -5, -6, -7 and -8). B: Densitometric analysis of the blots in A performed using Quantity One. Protein loading was evaluated by the GAPDH and actin band. Data are indicated as mean±SD. Significance was determined by one-tailed unpaired t-test. The level of significance was set at P<0.05. B: Relative mRNA levels of VCP and linkers between UPS and autophagy in normal and GNE myopathy patient biopsies. A total of four samples for each group were used, and each sample was run in triplicate for real-time PCR. C = control tissue, n = 4 (Samples are from control-1, -3, -4 and -5); GNE myopathy, n = 4 (Samples are from HIBM-5, -6, -7 and -8). Relative mRNA levels of VCP and linkers were increased in GNE myopathy, VCP to 215.2%, HDAC to 135.4%, NBR1 to 95.1% and p62 to 64.5%. Data are shown as means ± SD. The level of significance was set at P<0.05. The densitometry graphs are representative of only the one chosen in the corresponding blot.

Expression of VCP and linkers between UPS and autophagy by immunoblotting was increased in GNE myopathy muscle biopsies as compared to control biopsies (Fig. 5A). VCP was increased by 139.7%, HDAC6 by 98.6%, p62 by 124.8% and NBR1 by 149.2% (P<0.05) in GNE myopathy patients (Fig. 5B). Real-time RT-PCR analysis showed similar results (Fig. 5C).

## Discussion

GNE myopathy is a degenerative myopathy in which rimmed vacuole formation induces muscle fiber atrophy and loss. Mutations in the *GNE* gene are associated with GNE myopathy. They cause decreased GNE enzymatic activities resulting in decreased production of sialic acid, which then leads to the muscle degeneration that is characterized by rimmed vacuole formation and intranuclear filamentous inclusions in GNE myopathy. Several hypotheses about pathogenic mechanism of GNE myopathy have been proposed [3], [8], [15]. Malicdan has observed intracelllular amyloid deposition before the autophagy process in a mouse model of GNE myopathy [16]. Kumamoto suggested that ubiquitin proteasome proteolytic pathway as well as the nonlysosomal calpain and lysosomal proteolytic pathway may participate in the muscle fiber degradation in GNE myopathy [17]. Nevertheless, how these proteolytic pathways are initiated and what role they play in GNE myopathy pathogenesis are not fully understood.

As a glycoprotein, hyposialylation of Aβ is likely to lead to protein misfolding and the misfolded proteins may aggregate in the endoplasmic reticulum. Intracellular protein accumulation (mostly Aβ) is one of the most prominent characteristic features of sIBM and GNE myopathy [18]. The abnormal accumulation of AβPP and p-tau and its secondary responses such as UPR and the inhibition of the 26S proteasome in sIBM has been investigated [14], [19]. The two diseases share certain degree of similarity in term of clinical manifestations and pathological changes in muscle. To our knowledge, there is no study on whether the above pathological events also exist in GNE myopathy. In the present study, we observed round-shaped inclusions strongly immunoreactive to AβPP in normal-appearing muscle fibers in GNE myopathy patients. This suggests that AβPP accumulation precedes rimmed vacuole formation and muscle fiber atrophy, which is consistent with observations in a GNE myopathy mouse model [20]. We consider the abnormal protein accumulation as a general upstream event contributing to the pathogenic cascade of GNE myopathy. So we investigated the possible secondary responses to abnormal protein aggregates accumulation in GNE myopathy muscles. Accumulation of unfolded proteins in the endoplasmic reticulum can disturb endoplasmic reticulum functions and cause ERS. For survival, cells have developed an evolutionarily conserved adaptive response UPR to attenuate the protein synthesis, upregulate the transcription and translation of chaperone genes that increase endoplasmic reticulum capacity of protein processing, and retro-translocate misfolded proteins to the cytosol for degradation [21], [22]. We find that the levels of a set of ER chaperones, GRP94, GRP78, calnexin and calreticulin, but not ERp72, are increased and these proteins are multifocally accumulated in GNE myopathy muscle fibers, where they co-localize with AβPP, GRP78 and GRP94 form an endoplasmic reticulum chaperoning network with a set of endoplasmic reticulum molecular chaperones processing the unfolded protein substrates [23], [24], and the calnexin/calreticulin system recognize the nascent protein with monoglucosylated N-linked glycans for the subsequent folding and assembly steps. The upregulated chaperones may play a role in folding and retaining the terminally misfolded proteins in soluble conformations and preventing their aggregation in the ER lumen in GNE myopathy. We propose that UPR is part of the GNE myopathy pathogenic cascade, occurring in response to abnormally unfolded or misfolded proteins, and that UPR is an attempt to facilitate the proper folding and/or disposal of misfolded proteins.

When the protective effect of UPR fails to overcome the stress situation, endoplasmic reticulum employs ERAD (endoplasmic reticulum associated degradation) to clear the aggregated misfolded or unassembled proteins. During ERAD, the target proteins selected by endoplasmic reticulum quality control system are retro-translocated to the cytosol by a cytosolic AAA-ATPase VCP (also called Cdc48/p97) and degraded by the ubiquitin–proteasome system [25], [26]. Whether such ERAD mechanism exists in GNE myopathy is unknown. In our study, abundant VCP in muscle tissue from GNE myopathy patient indicated an enhanced delivery process of misfolded proteins from endoplasmic reticulum to cytosol.We also found significantly increased activities of three major proteasomal proteolytic enzymes accompanied by the up-regulation of 20S proteasome subunits both on protein and mRNA levels, indicating UPS is activated to eliminate unfolded proteins in GNE myopathy muscle.

The autophagy-lysosomal system and ubiquitin-proteasome system are the two major pathways that accomplish protein catabolism involved in most aspects of normal physiology and development, and in a broad array of pathological states. Autophagy is mainly responsible for the degradation of long-lived proteins and cytoplasmic organelles in eukaryotic cells. The UPS and autophagy were, for a long time, regarded as independent degradative pathways with little or no interaction. This view has started to change recently and the parallels between UPS and autophagy in their roles and regulation has been highlighted [27], [28]. Although UPS activation was confirmed in our study and the autophagic nature of these vacuoles is supported by the observations of acid-phosphatase rich primary lysosomes, clathrin-positive granules, and the presence of cathepsins B and L in GNE myopathy [29], how the two proteolytic pathways are regulated is still an unsolved problem. There are several proteins that appear to serve as linkers between ubiquitinated cargo and the phagophore, including p62, NBR1 and HDAC6 [30]–[32]. These proteins have the capacity to interact directly or indirectly with both ubiquitin and components of autophagic machinery, thus being a suitable link as an adaptor molecule. And some of these linker proteins such as NBR1 and p62 have been recently found to be increased and involved in protein degradation pathway in sIBM [33], [34]. Here we found the linkers between UPS and autophagy were abundantly expressed in GNE myopathy patients. The increase of those linker proteins between UPS and autophagy manifest an enhanced delivery of ubiquitinated unfolded/misfolded proteins from UPS to autophagy system. In GNE myopathy, excessive autophagy should be the result of accumulation of abnormal proteins which are not successfully eliminated by cytosolic proteases. Protein aggregation then ensues, in which HDAC6 might play a role. However, aggregation makes these proteins ostensibly resistant to cytosolic proteases, leaving autophagy as the only viable compensatory possibility for their removal.

Our study indicates that the proteosomal system is activated to participate in ERAD during ERS resulted from Aβ deposition in GNE myopathy. Highly expressed VCP enhances the delivery of unfolded proteins from endoplasmic reticulum to proteosomal system. However persistent abnormal unfolded proteins accumulation probably exceeded the capability of UPS degradation. So excessive ubiquitinated unfolded proteins are exported to autophagy system by linkers between UPS and autophagy, which is activated as an alternative pathway for degradation.

## Supporting Information

Table S1 **Clinical characteristics of individual GNE myopathy patients and controls.**
(DOCX)

Table S2 **Various antibodies used in immunohistochemical reactions and immunoblotting in GNE myopathy muscle fibers.**
(DOCX)

Table S3 **Primers sequences used in relative-quantitative real-time RT-PCR.**
(DOCX)
