# Supplementary material for: Unfolded Protein Response and Activated Degradative Pathways Regulation in GNE Myopathy
Source: PLoS One. 2013 Mar 5;8(3):e58116. doi: 10.1371/journal.pone.0058116 (PMC3589370; doi:10.1371/journal.pone.0058116)
Supplement: Table S1 — Clinical characteristics of individual GNE myopathy patients and controls. (DOCX) [file pone.0058116.s001.docx]

Table S1 - Clinical characteristics of individual GNE myopathy patients and controls

|  | **Sex** | **Age**  **(y)** | **Disease duration**  **(y)** | **Biopsy**  **site** | **Pathology findings** | | **CK (U/L)** | **EMG** | **GNE mutation/**  **Final diagnosis** |
| --- | --- | --- | --- | --- | --- | --- | --- | --- | --- |
|  |  |  |  |  | **RVs** | **Necrosis** |  |  |  |
| patient-1 | F | 25 | 3 | BB | 12.5% | A few | 213 | M | p.L508S/p.A631V |
| patient-2 | F | 23 | 2 | BB | 13.8% | - | <200 | M/N | p.L508S/p.A631V |
| patient-3 | F | 30 | 11 | BB | 34.8% | - | 307 | N | p.I51M/p.L508S |
| patient-4 | F | 23 | 1 | BB | 0.24% | - | ND | N | p.E35K/p.L508S |
| patient-5 | F | 27 | 7 | BB | 16.8% | - | <200 | M | p.D176V/p.L508S |
| patient-6 | F | 32 | 7 | BB | 37.2% | - | 622 | M | p.I587T/p. I587 |
| patient-7 | F | 24 | 4 | BB | 14.3% | - | 513 | N | p.Y166H/p.D515fsX2 |
| patient-8 | M | 25 | 1 | BB | 7.2% | Some | 444 | M | p.D176V/p.V305F |
| Control-1 | F | 33 | 1/4 | BB | Normal | | 876 | N | Primary epilepsy |
| Control-2 | F | 28 | 1/12 | BB | Normal | | ND | ND | Osteosarcoma |
| Control-3 | F | 23 | 1/12 | BB | Normal | | ND | ND | Primary epilepsy |
| Control-4 | F | 38 | 1/12 | BB | Normal | | 33 | ND | Primary epilepsy |
| Control-5 | M | 26 | 1/12 | GC | Normal | | 2402 | ND | Primary epilepsy |

Abbreviations: RV, rimmed vacuoles; BB, biceps brachii; GC, gastrocnemius; CK, creatine kinase; EMG, electromyogram; M, myogenic patterns (reduced amplitude and duration of motor unit action potential), N, neurogenic pattern (reduced motor nerve conduction velocity or sensory nerve conduction velocity; autonomous potential); ND, not done;
