# Supplementary material for: Unfolded Protein Response and Activated Degradative Pathways Regulation in GNE Myopathy
Source: PLoS One. 2013 Mar 5;8(3):e58116. doi: 10.1371/journal.pone.0058116 (PMC3589370; doi:10.1371/journal.pone.0058116)
Supplement: Table S2 — Various antibodies used in immunohistochemical reactions and immunoblotting in GNE myopathy muscle fibers. (DOCX) [file pone.0058116.s002.docx]

Table S2. Various antibodies used in immunohistochemical reactions and immunoblotting in GNE myopathy muscle fibers

| Antibodies Type Corporation | | |
| --- | --- | --- |
| β-APP  P-tau  GRP94  ERp72  GRP78  Calnexin  Calreticulin  Caspase12  Caspase3  Proteasome subunitα2  Proteasome subunitα4  Proteasome subunitβ5  HDAC6  NBR1  SQSTM1(p62)  VCP | mouse monoclonal  rabbit polyclonal  rabbit polyclonal  rabbit polyclonal  mouse monoclonal  mouse monoclonal  mouse monoclonal  rabbit polyclonal  rabbit polyclonal  mouse monoclonal  mouse monoclonal  mouse monoclonal  mouse monoclonal  mouse monoclonal  mouse monoclonal  mouse monoclonal | Abcam ab78271  Santa Cruz Biotechnology sc-32828  Abcam ab18055  Abcam ab11422  BD Transduction Laboratories 610978  BD Transduction Laboratories 610523  BD Transduction Laboratories 612137  Abcam ab18766  Abcam ab32351  Santa Cruz Biotechnology sc-67339  Santa Cruz Biotechnology sc-58415  Santa Cruz Biotechnology sc-55009  Santa Cruz Biotechnology sc-28386  Abcam ab55474  Santa Cruz Biotechnology sc-28359  Santa Cruz Biotechnology sc-133125 |
