# Supplementary material for: Unfolded Protein Response and Activated Degradative Pathways Regulation in GNE Myopathy
Source: PLoS One. 2013 Mar 5;8(3):e58116. doi: 10.1371/journal.pone.0058116 (PMC3589370; doi:10.1371/journal.pone.0058116)
Supplement: Table S3 — Primers sequences used in relative-quantitative real-time RT-PCR. (DOCX) [file pone.0058116.s003.docx]

Table S3. Primers sequences used in relative-quantitative real-time RT-PCR

| Sequence no. | Gene name | Primer sequences | Accession no. | Tm(℃) | Product size(bp) |
| --- | --- | --- | --- | --- | --- |
| 1  2  3  4  5  6  7  8  9  10  11  12 | GRP78  GRP94  ERp72  Calnexin  Calreticulin  Proteasome subunitα2  Proteasome subunitα4  Proteasome subunitβ5  HDAC6  NBR1  P62  VCP | F- TCAAGTTCTTGCCGTTCAAGG  R- AAATAAGCCTCAGCGGTTTCTT  F- CCAGTTTGGTGTCGGTTTCTAT  R- CTGGGTATCGTTGTTGTGTTTTG  F- GGCTGACAAAGATACAGTGCT  R- GTGGGGTAGCCACTCACAT  F-TCCATGACAAGACCCCTTATACG  R- GTGCAGTTTATAGTCCTCTCCAC  F- AGTTCCGGCAAGTTCTACGG  R- ACAGAGCATAAAAGCGTGCAT  F- GCAATGAAAAGATCGTGGTCCT  R- GAAGCCTTCTAGCTTGGTGTG  F- AGTGTGGCAGGCATAACTTCT  R- TCACAAGGTATTGGCTCCTGA  F- AGGAACGCATCTCTGTAGCAG  R- AGGGCCTCTCTTATCCCAGC  F- CAACTGAGACCGTGGAGAG  R- CCTGTGCGAGACTGTAGC  F- CTCTGCATACAAGGCCCTGTT  R- CTTCAGCAGCCGTAGGTTCAG  F- CCAGTGACGAGGAATTGACAA  R- CATCGCAGATCACATTGGGG  F- GACACAGTGTTGCTGAAAGGA  R- TTCCGAACAACTCTATTCATCCG | NM_005347  NM_003299  NM_009787  NM_001024649  NM_004343  NM_176783  NM_002789  NM_001144932  NM_006044  NM_005899  NM_003900  NM_007126 | 60  60  60  60  61  60  60  62  61  62  60  60 | 148  82  189  68  79  194  101  116  108  139  156  107 |
